# Narcolepsy or Sleep Apnea?: Not Everything is What it Appears to be!

**DOI:** 10.3389/fneur.2013.00152

**Published:** 2013-10-08

**Authors:** Seithikurippu R. Pandi-Perumal, Ravi Gupta, Jaime M. Monti

**Affiliations:** ^1^Somnogen Canada Inc., Toronto, ON, Canada; ^2^Department of Psychiatry and Sleep Clinic, Himalayan Institute of Medical Sciences, Dehradun, India; ^3^Department of Pharmacology and Therapeutics, School of Medicine, Clinics Hospital, Montevideo, Uruguay

**Keywords:** excessive daytime sleepiness, elderly, narcolepsy, rapid eye movement, multiple sleep latency test, sleep apnea, sleep onset REM periods, epworth sleepiness scale

The world is seldom what it seems; to man, who dimly sees, realities appear as dreams, and dreams realities.

–Samuel Johnson

We are reluctant to intrude in a discussion in this case report. Based on their clinical observations of the patient (an 84-year-old woman with long-standing history of excessive daytime sleepiness (EDS), the study authors’ most likely diagnosis was late onset narcolepsy. However, we believe that some of the inferences made by Carlucci and Prasad (1) merit further comments.

We see this as an interesting and important case report and professionally reported. However, we view the number of atypical symptoms and their significance from a perspective that is somewhat different from those of the authors. We believe that there are several fundamental questions that need to be addressed here. In areas where we do not agree with the authors, we have stated them with the rationale for our disagreement. We address our comments point by point as follows:

The patient was reported to be morbidly obese (BMI = 36), and to have a large neck circumference (NC = 17), and a Mallampatti Gr III airway. Nevertheless the patient had a normal AHI which was just 0.9 with normal overnight oximetry. Further, the overnight hypnogram reveals that most, if not all, apneas are rapid eye movement (REM) related, but not positional. Although not impossible, the inference that the apneas were almost exclusively a REM sleep phenomenon appears improbable. The first question is – was the observed low AHI related to the poor quality of sleep on the testing nights (sleep efficiency 64 and 71%).

Second, this lady was not described having any other symptoms that had supported the diagnosis of narcolepsy viz., hypnagogic or hypnopompic hallucination. If they were present, their presence should be considered in presence of poor quality sleep in night, as recorded by two overnight polysomnograms.

Third, the patient was described as excessively sleepy (ESS score: 11/24). The cut-off ESS score is an arbitrary one and based upon the population norm. Our concern is can a patient be categorized as excessively sleepy because she scored just 1 point more than the population norm, i.e., 10. We think, an important issue to consider is the compromise in daytime functioning because of sleepiness, especially when we are dealing with a single case. Moreover, the authors state that at the time of the study she was on multiple medicines including cyclobenzaprine, and hydrocodone, which were taken as needed. Hydrocodone and cyclobenzaprine both can produce drowsiness. Hence, ingestion of these medications should also have been taken into consideration while inferring the information about EDS. The report mentions that patient had goiter. The question is, whether the thyroid profile was within normal limits? Moreover, was the toxicology screening done to find out the reason for excessive daytime sleepiness?

Fourth, was the quality and duration of nighttime sleep sufficient to justify performing an MSLT? (2). The inadequate sleep that the patient had the night before could also have resulted in daytime sleepiness and SOREMPs (3).

Fifth, as per self-reports of the patient, the authors mentioned that she maintained a regular schedule (although no mention was made with regards to her usual bed time). Considering her age, is it possible that her habitual time of retiring was much earlier than the sleep time followed in laboratory (11:02 p.m. as per the study record); this could have produced an early onset of REM during the overnight polysomnogram.

Sixth, as per the hypnogram, the patient had long period of self-awakenings from all of her REM periods. The interesting question is – would this have produced early REMs in her MSLT, especially during first MSLT session during the subsequent daytime study? For example, REM sleep onset latency (REMOL) in the first REM period in the MSLT was markedly reduced (3 min) while subsequent MSLT tests showed somewhat delayed REMOL. Another possibility when we see this data in association with poor sleep efficiency is that the patient might have been almost, if not already sleeping during the hookup of the first MSLT. Only a clear video recording review would prove this. It should be considered also that severe or complex psychiatric, neurological, or medical disorders, and the use of anti-cataplectic or stimulants can compromise the validity of the MSLT. In addition, sleep latency on MSLT and number of SOREMPs usually decreases as a function of age (4).

Seventh, after 7 h on the study night, the patient was awakened during her REM period. We suggest that brief notations from the patient’s sleep diary would have been helpful for confirming the validity of the authors’ conclusions, i.e., what was her typical bedtime routine, e.g., time to bed, total time in bed, subjective measure of how long she took to fall asleep, how long she slept, subjective feeling of how she felt about her night sleep, i.e., of whether she felt feeling refreshed or not) and so on. There is insufficient data to rule out chronic sleep deprivation in this patient and hence, MSLT alone cannot be relied to make a diagnosis of narcolepsy (5, 6).

Eighth, an N3 of 24% at the age of 84 years appears atypical (7).

Ninth, we also found some striking abnormalities in the cardiorespiratory events during the second REM cycle (between 12:46 and 1:46 a.m.). Our visual analysis shows a surge in the heart rate dynamics as well as changes in the snoring pattern. These changes in presence of snoring suggest presence of sleep related breathing disorder.

Tenth, can the falls described in this case be interpreted as cataplexy at the age of 84 years? Cataplexy has been considered by many authors as the most accurate diagnostic marker of the disease. Studies have shown that 65–75% of patients with narcolepsy have cataplexy (8). Cataplexy worsens with poor sleep and fatigue and often improves with advancing age. The majority of patients begin to show symptoms in the second decade of life, and the distribution is bimodal, with a large peak around puberty and a smaller peak between 35 and 45 years of age (9). Almost all patients with typical cataplexy are positive for HLA-DQBI*06.02. The HLA typing was not done even though it had a reasonable chance of being positive (10). Additionally, we believe that other potential contributors to the patient’s “*cataplectic episodes*” are possible, especially in the context of the patient’s multiple comorbidities and polypharmacy (10).

Eleventh, we did not find any mention of measurement of hypocretin (orexin)-1 levels in the CSF. Levels of orexin-1 should be lower than 110 pg/ml. Low CSF levels of orexin are included in the diagnostic criteria for narcolepsy of the second revision of the International Classification of Sleep Disorders (11).

Lastly, in clinical practice, narcolepsy occurrence is rare in old age. So we have to be cautious in making a diagnosis of narcolepsy in the elderly (11, 4).

In short, based on the presence of excessive daytime sleepiness (EDS), sleep paralysis, and sleep onset rapid eye movement periods (SOREMPs) as evident from the MSLT tests, one could persuasively argue that the patient in question might have a late onset narcolepsy. Additional support also could come from the increased incidence of sleep disordered breathing among narcoleptics. The prevalence of sleepiness is rather not uncommon in a number of medical illnesses including primary and co-morbid sleep disorders. In clinical practice, whether it is a co-morbid condition or a secondary symptom is often misdiagnosed (12). Excessive daytime sleepiness (EDS) can also exist as an independent disorder despite its co-occurrence with other illnesses such as narcolepsy, sleep apnea. Although sleep onset REM periods (SOREMPs) in two or more of naps based on MSLT findings is highly suggestive of a diagnosis of narcolepsy; one could also speculate that there are other medical conditions, which can mimic narcolepsy, such as sleep apnea and severe sleep deprivation.

Any person with EDS can have SOREMPs (e.g. shift workers, patient’s with sleep apnea, and a wide variety of sleep and psychiatric disorders (13–15). Yet, the diagnosis of narcolepsy can be certain, only if all other probable causes can be ruled out. Despite these reasons, other than narcolepsy, sleep paralysis can occur in isolation or can be linked to disorders such as migraines, anxiety disorders, and OSA. Although, high prevalence of narcolepsy in conjunction with sleep paralysis, ruling out other potential sleep disorders that could account for the feelings of paralysis is mandatory.We have reviewed the evidence presented in this case. We find the article was interesting but unfortunately superficial, neglected a thorough EDS differential screen and work-up that they mention but reject: diary, toxicology screen, and TSH for goiter, thereby making the authors’ diagnosis questionable based on the information provided in their case report.

Given these differences in our opinion and considering the reasons that we have given, we would have been very cautious in labeling this case as narcolepsy unless all other confounding variables were also considered. Further investigations to update these strategies are required for an efficient management of this patient for a better therapeutic response.

We wish the authors take our views as a constructive criticism of their report.

## Disclosure Statement

The authors have read the journal’s policy and have the following potential conflicts: S.R. Pandi-Perumal is a stockholder and the President and Chief Executive Officer of Somnogen Canada Inc., a Canadian Corporation. He declares that he has no competing interests that might be perceived to influence the content of this article. This does not alter the authors’ adherence to all the journal policies. All remaining authors declare that they have no proprietary, financial, professional, nor any other personal interest of any nature or kind in any product or services and/or company that could be construed or considered to be a potential conflict of interest that might have influenced the views expressed in this manuscript.
